# Bioresorbable scaffold implantation in STEMI patients: 5 years imaging subanalysis of PRAGUE-19 study

**DOI:** 10.1186/s12967-020-02230-1

**Published:** 2020-01-30

**Authors:** Viktor Kočka, Petr Toušek, Martin Kozel, Andrea Buono, Martin Hajšl, Libor Lisa, Tomáš Buděšínský, Martin Malý, Petr Widimský

**Affiliations:** 1 Cardiocenter, Third Faculty of Medicine, Charles University and University Hospital Kralovské Vinohrady, Šrobárova 50, Prague 10, 100 34 Czech Republic; 2 Department of Cardiology, Niguarda Ca’ Granda Hospital, Milan, Italy; 3 Cardiovascular Center, First Faculty of Medicine, Charles University and Central Military Hospital Prague, Prague, Czech Republic

**Keywords:** Bioresorbable scaffold, STEMI, Long-term follow-up, Quantitative coronary angiography, Optical coherence tomography

## Abstract

**Background:**

Bioresorbable scaffold (BRS) Absorb™ clinical use has been stopped due to higher rate of device thrombosis. Scaffold struts persist longer than 2 years in the vessel wall. Second generation devices are being developed. This study evaluates long-term invasive imaging in STEMI patients.

**Methods:**

PRAGUE-19 study is an academic study enrolling consecutive STEMI patients with intention to implant Absorb™ BRS. A total of 83 STEMI patients between December 2012 and March 2014 fulfilled entry criteria. Coronary angiography and optical coherence tomography at 5 year follow-up was performed in 25 patients.

**Results:**

Primary combined clinical endpoint (death, myocardial infarction or target vessel revascularization) occurred in 12.6% during the five-year follow-up with overall mortality 6.3%. Definite scaffold thrombosis occurred in 2 patients in the early phase after BRS implantation. Quantitative coronary angiography after 5 years demonstrated low late lumen loss of 0.11 ± 0.35 mm with binary restenosis rate of 0%. Optical coherence tomography demonstrated complete resorption of scaffold struts and mean lumen diameter of 3.25 ± 0.30 and 3.22 ± 0.49 (P = 0.73) at baseline and after 5 years, respectively. Three patients developed small coronary artery aneurysm in the treated segment.

**Conclusion:**

Invasive imaging results 5 years after BRS implantation in STEMI showed complete resorption of scaffold struts and stable lumen vessel diameter.

*Trial registration* ISRCTN43696201 (retrospectivelly registred, June 7th, 2019). https://www.isrctn.com/ISRCTN43696201.

## Background

Bioresorbable scaffolds (BRS) were developed to provide mechanical support and anti-restenotic properties in the short term and then undergo resorption, thus avoiding the permanent metallic caging of treated coronary artery. The Absorb™ device (Abbott Vascular, Santa Clara, CA, USA) is by far the most studied BRS to date. The initial 1-year results were encouraging but three meta-analyses demonstrated that Absorb BRS is associated with increased risk of stent thrombosis, myocardial infarction (MI) and target lesion failure at 2–3 years post implantation [1–3]. As a result, the commercial use of Absorb™ BRS has stopped in 2017. The recently published ESC guidelines on myocardial revascularization recommend using any other BRS only in carefully controlled clinical studies [4]. However, the recently published largest randomized ABSORB IV study of Absorb™ BRS (not included in the above cited meta-analyses) with optimal implantation technique and careful patient selection resulted in non-inferior target lesion failure at 1 year [5]. Thus, the discussion about safety and effectivity of BRS technology continues. It is vital for the possible future development of next generation BRS devices to demonstrate safety and efficacy after the completion of resorption. Up to date, 5 years clinical and imaging data are available only from Absorb A and Absorb B stable patients’ cohorts [6–8]. We therefore sought to study 5 year invasive imaging data of patients with BRS implantation in the setting of ST-elevation myocardial infarction (STEMI).

## Methods

### Study population

The PRAGUE-19 study is a prospective two-centre open-label registry of consecutive STEMI patients. One hundred and thirty-four consecutive STEMI patients fulfilled the inclusion criteria and were treated with Absorb™ BRS version 1.1 during the enrolment period between December 2012 and December 2015. The design, short-term results of the pilot study phase and subsequently interim analysis of three years clinical and imaging results have been published previously [9, 10]. This article represents the imaging analysis of the first 25 patients available for the 5 year invasive assessment as planned in the study protocol and approved by the ethical committee. Article also presents interim analysis of five-year clinical follow-up of patients enrolled from December 2012 until March 2014, this study cohort is available for five-year outcome assessment in March 2019. During this period, 83 patients fulfilled the inclusion criteria for Absorb™ BRS implantation; this device was successfully implanted in 81 (97%) patients. In two patients, Absorb™ BRS could not be delivered to the culprit lesion and a metallic stent was used instead. The study protocol mandated dual antiplatelet therapy for 12 months after Absorb™ BRS implantation and all our patients adhered to this protocol. The study was approved by the local ethics committee at each centre as well as by the national multicentric ethics committee, and written informed consent was obtained from all study patients. The originally planned coronary angiography with optical coherence tomography (OCT) at three-year follow-up was postponed to five years after analysis of three-year results demonstrating incomplete resorption of the Absorb™ BRS device [10]; study protocol was amended and invasive assessment was approved for the first 25 patients by all ethics committees again. Complete clinical follow-up of all patients included in the study as well as patients in the control group is planned and will be finished in December 2020. This study was conducted according to the Declaration of Helsinki.

### Clinical and imaging 5-year follow-up

**Fig. 1:**
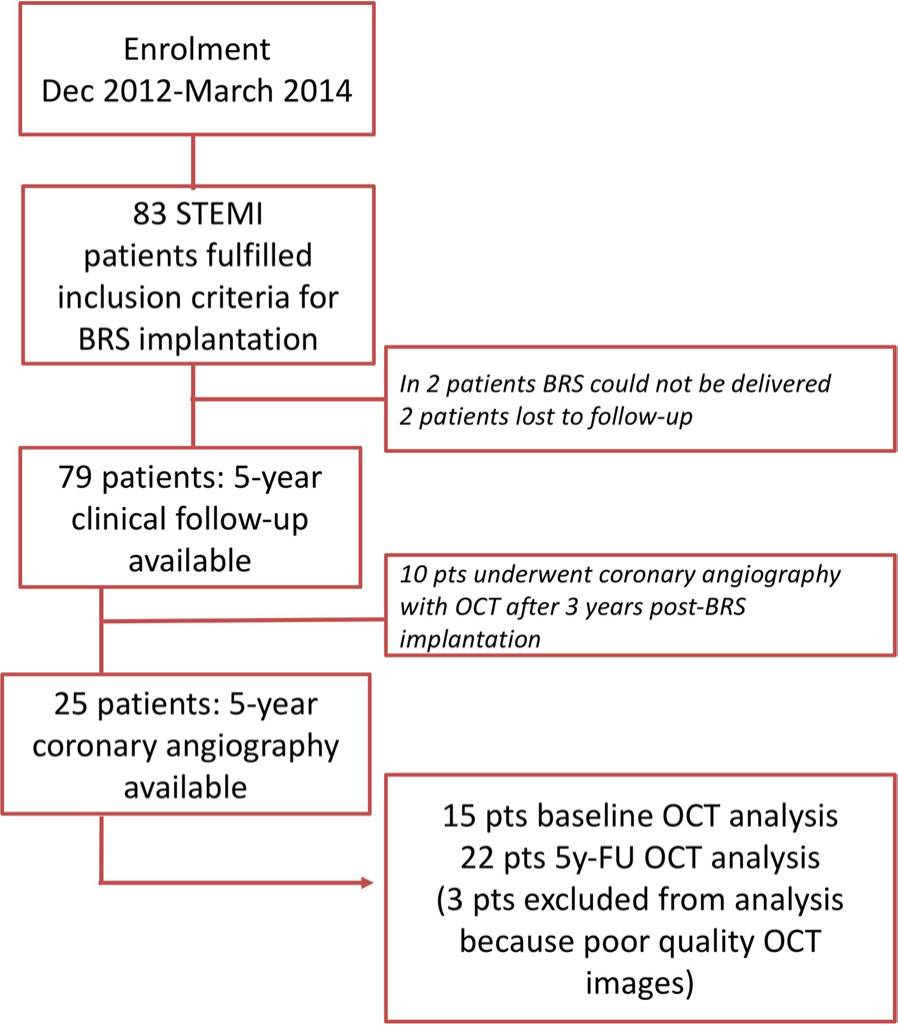
Study flow chart. BRS: bioresorbable scaffold; FU: follow-up; OCT: optical coherence tomography; STEMI: ST-elevation myocardial infarction

The 5-year clinical follow-up was evaluated in 79 of 81 patients with implanted Absorb™ BRS (two patients were lost to follow-up). The study flow chart is presented in Fig. 1. The prespecified primary endpoint of the study was a combination of death, MI and target vessel revascularization (TVR). BRS thrombosis was defined according to the Academic Research Consortium definitions [11]. All patients had quantitative coronary angiography (QCA) off-line analysis of angiogram after completion of primary percutaneous coronary intervention (PCI). Optical coherence tomography in the acute phase at the end of primary PCI was recommended but not mandatory (the most common reason not to perform OCT was patient hemodynamic instability or clinically significant arrhythmia). Invasive coronary imaging at five years was performed only in patients who agreed with the research procedure and did not undergo repeat coronary angiography at three-year follow-up. All invasive studies at five-year follow-up included OCT. Intracoronary nitrate has been injected into all studied arteries prior to imaging. All procedures were performed via radial artery approach without any complications, most patients were discharged on the same day.

### QCA analysis

For each angiogram, QCA was performed using Philips Xcelera software (Philips Medical Systems, Eindhoven, The Netherlands). Identical or at least similar projections were used for comparison of baseline study at the end of primary PCI with the five-year follow-up study. The treated segment was defined as 5 mm proximal and distal to the visible proximal and distal Absorb™ BRS markers. Image calibration was performed with a contrast-filled 6 French guiding catheters. The following QCA parameters were measured: proximal and distal reference vessel diameter (RVD), minimal lumen diameter (MLD), mean lumen diameter, scaffold length between the 2 markers, Mean RVD was calculated as average of proximal and distal RVD. Diameter stenosis was calculated from the MLD and mean RVD and expressed as a percentage. Late lumen loss was defined as MLD at five-year follow-up angiography minus MLD at baseline study at the end of primary PCI. The presence of coronary artery aneurysm was defined by focal vessel dilatation with diameter at least 50% larger than the adjacent reference segment [12].

### OCT acquisition and analysis

**Fig. 2:**
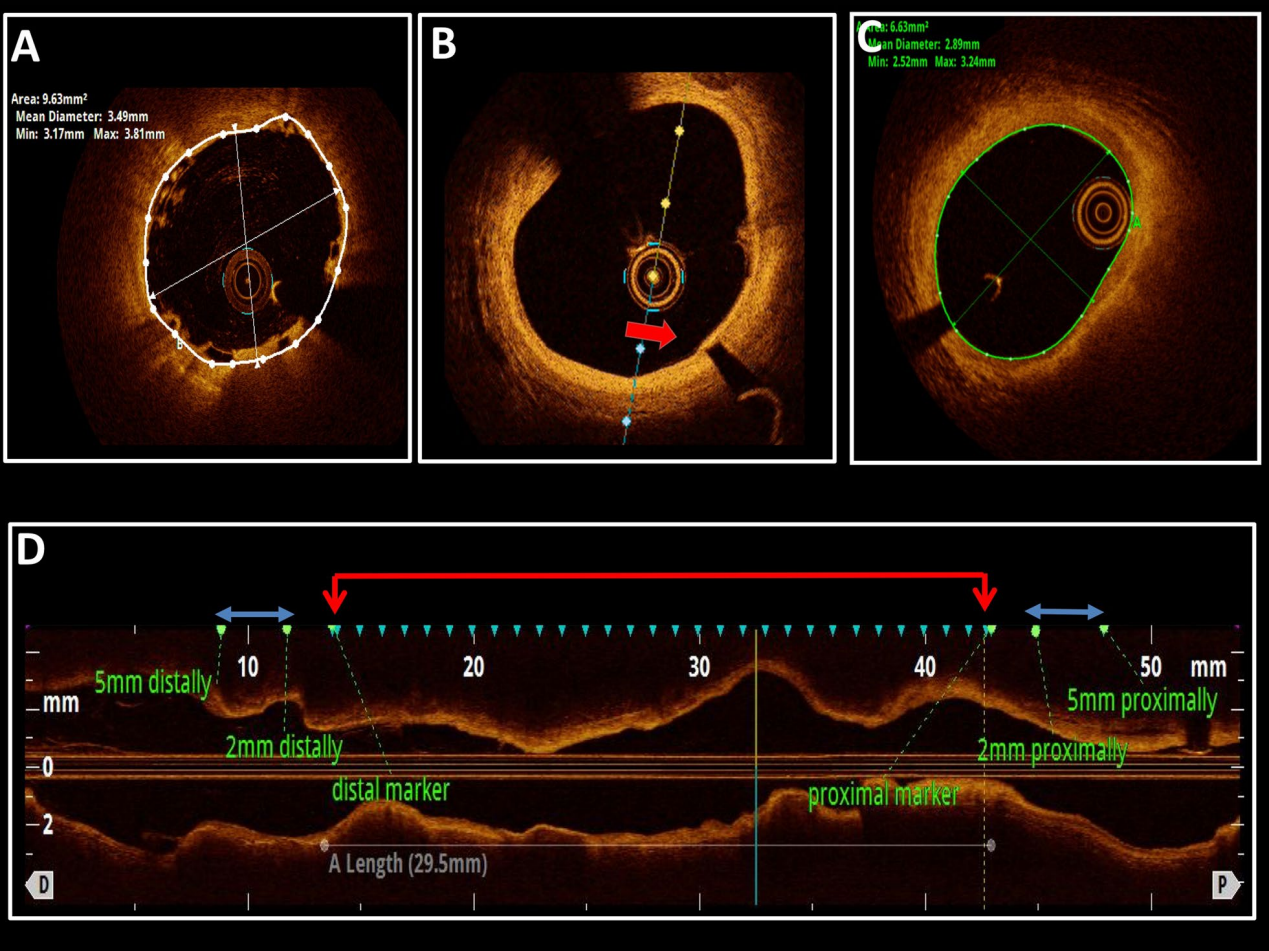
Description of OCT methodology. **A** In-scaffold area (white line) and diameter calculation immediately after Absorb BRS implantation. Note inclusion of scaffold struts (box-like structures) into the measured area. **B** scaffold marker identified with typical shadow (red arrow). **C** lumen area (green line) and diameter calculation at five-year follow-up. Note complete absence of residual scaffold struts. **D** longitudinal view of vessel segment assessed by OCT. After proximal and distal stent marker identification (red arrows), in-scaffold and lumen areas and diameters have been calculated in 1 mm cuts mm (represented by blue triangles). Proximal and distal vessel reference areas and diameters have been calculated as the mean values between 2 and 5 mm (represented by blue lines with arrows). The same methodology has been applied at baseline and five-year follow-up.* BRS* bioresorbable scaffold,* OCT* optical coherence tomography

Technique of OCT acquisition at the end of primary PCI has been described previously [9]. OCT at five-year follow-up was performed using the frequency domain C7 system with a Dragonfly Duo catheter (St. Jude Medical, St. Paul, MN, USA), a pullback speed of 20 mm/sec, and an image acquisition of 100 frames/sec. OCT off-line analysis was performed using the proprietary software (ILUMIEN OPTIS system, Light Lab Imaging, Westford, MA, USA) which has a validated reproducibility [13]. All OCT measurements were based on previously published standards [14]. However, some adjustments were required due to Absorb™ BRS complete resorption at five years post implantation. At first, proximal and distal scaffold markers were identified on the OCT pullback. The design of Absorb™ BRS is such, that the scaffold edges extend beyond the markers by 0.3 mm at the distal end and by 0.9–1.3 mm at the proximal end [15]. We have therefore measured proximal and distal reference vessel area and mean diameter as a mean value of frames at the distance between 2 and 5 mm from the scaffold markers (Fig. 2). Scaffold area/diameter (baseline OCT) and lumen area/diameter (five-year OCT) were measured at vessel cross-sections at 1 mm intervals. Mean scaffold/lumen area and diameter were calculated as mean of all-cross-sections between the scaffold markers. Minimal lumen area/diameter were measured at the site of the smallest lumen between the scaffold markers. It is not possible to measure neointimal hyperplasia area in the absence of stent/scaffold struts. The number of visible struts was noted in each 1 mm cut. Coronary artery dissection, the presence of thrombus and coronary artery aneurysm were all recorded in every patient.

### Statistical analysis

Standard descriptive statistics were applied in the analysis: absolute and relative frequencies for categorical variables; continuous variables were described using the mean supplemented with standard deviation. The statistical significance of changes in continuous variables (QCA and OCT) between baseline and 5-year follow-up was tested using a paired t-test. P-values < 0.05 were considered statistically significant. Statistical analysis was computed using SPSS 23.0.0.0 (IBM Corp., Armonk, NY, USA).

## Results

### Clinical follow-up

Baseline characteristics of 79 patients with implanted Absorb™ BRS are shown in Table 1. During the five-year follow-up period the primary endpoint (combination of death, MI and TVR) occurred in 10 patients (12.6%) with overall mortality of 6.3%. There were 3 cardiovascular deaths (all sudden deaths) at 14 days, 9 months and 51 months after Absorb™ BRS implantation. Two patients died due to cancer (brain and prostatic) at 40 and 50 months after BRS implantation. Two patients suffered of reinfarction (one subacute BRS thrombosis after ticagrelor discontinuation, one culprit lesion in different coronary artery tree) and three patients of TVR (one BRS restenosis and 2 native lesions not related to BRS segment) during the first 12 months. Except one sudden cardiac death and two non-cardiac deaths, there were no other clinical events after 12 months when dual antiplatelet therapy was stopped. All clinical events are summarized in Table 2.

**Table 1:** Baseline characteristics of patients with implanted Absorb BRS till March 2014 (N = 79)

Age, years (SD)	60 (11)
Male sex, n (%)	54 (68)
Diabetes mellitus, n (%)	7 (9)
History of smoking, n (%)	56 (71)
Infarct-related artery, n (%)	
Left anterior descending artery	35 (44)
Left circumflex artery	16 (20)
Right coronary artery	28 (35)
Left ventricle EF at discharge, % (SD)	53 (10)

*BRS* bioresorbable scaffold, *SD* standard deviation, *EF* ejection fraction

**Table 2:** Primary clinical endpoint (combination of death, MI and TVR)

	At 5 years FU (N = 79)	Years 1–5 (N = 77)
Primary clinical endpoint, n (%)	10 (12.6%)	3 (3,9%)
Death, n (%)	5 (6.3%)	3 (3.9%)
Cardiovascular (sudden death at 14 days, 9 and 51 months), n (%)	3 (3.8%)	1 (1.3%)
Non-cardiac (cancer at 40 months and 50 months), n (%)	2 (2.5%)	2 (2.6%)
Myocardial infarction, n (%)	2 (2.5%)	0 (0%)
Target vessel revascularization, n (%)	3 (3.8%)	0 (0%)
BRS thrombosis definite/probable, n (%)	2 (2.5%)	0 (0%)

*MI* myocardial infarction, *TVR* target vessel revascularization, *FU* follow-up, *BRS* bioresorbable scaffold

### Invasive assessment at 5-year follow-up

**Fig. 3:**
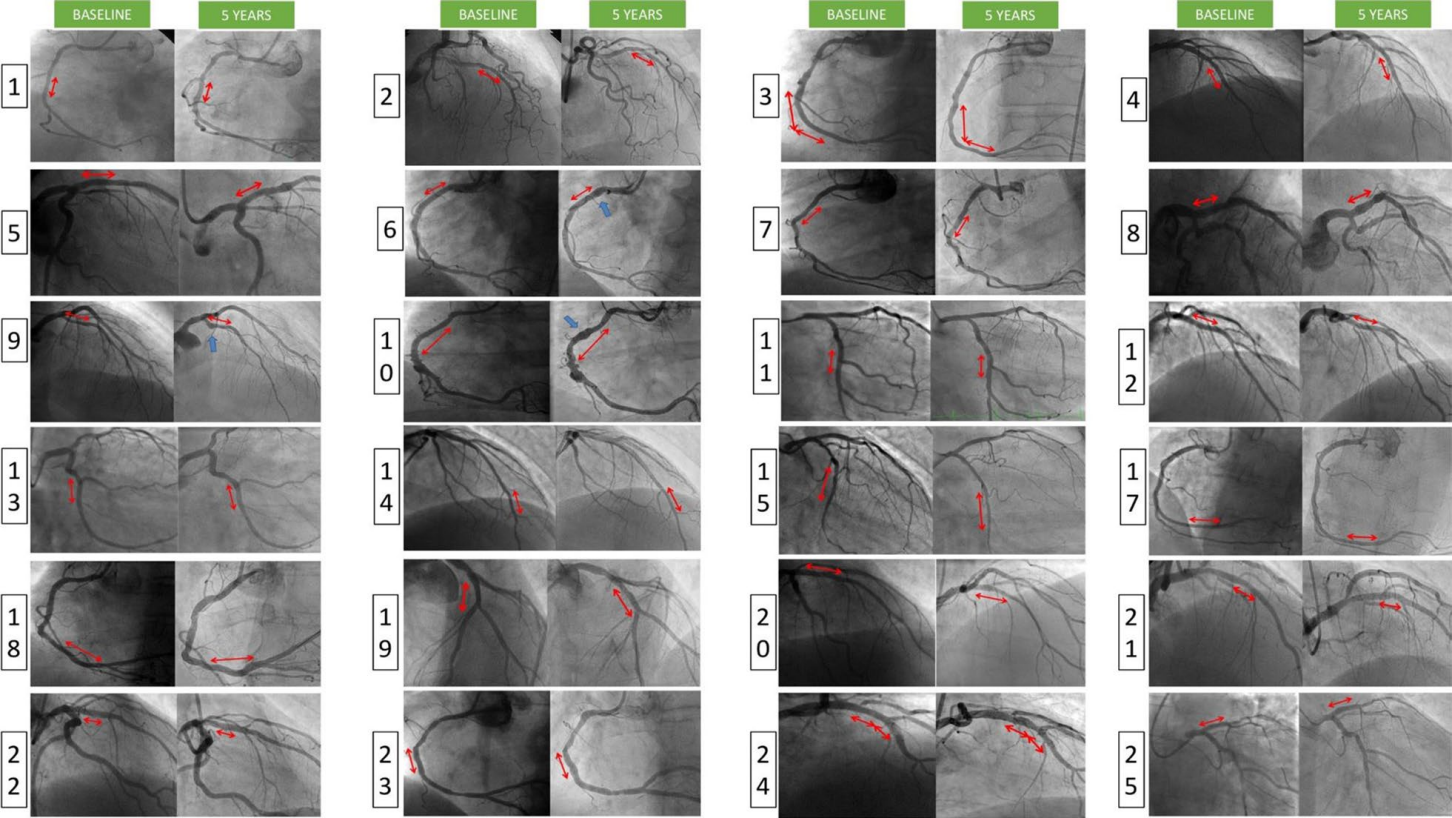
Paired analysis of coronary angiograms at baseline (left columns, acquired at the end of primary PCI) and after 5 years (right columns) in identical or similar fluoroscopic views. Patients are numbered. The only patient not presented here is Patient 16, who unfortunately did not have similar fluoroscopic views acquired, but for completeness the baseline and 5 years angiograms are presented in Supplement 1 online.* PCI* percutaneous coronary intervention

The results of QCA analysis performed at baseline and after five years are presented in Table 3. All patients had coronary angiograms available for analysis. There is no difference in scaffold length and reference vessel diameters. Late lumen loss was only 0.11 ± 0.35 mm after five years. There was no stenosis over 50% of diameter. Diameter stenosis was 15.6 ± 15.9% at baseline and 18.9 ± 8.3% (p = 0.05) after five years. Figure 3 shows coronary angiography of all patients with identical or similar fluoroscopic view at baseline and at five-year follow up (N = 24). One patient (No 16) did not have similar fluoroscopy views available and the coronary angiography in different views is available in online Supplement 1. Three patients developed new coronary artery aneurysm (patients 6, 9 and 10 in Fig. 3). All were small with maximum diameter between 50–100% larger than the adjacent reference vessel diameter.

**Table 3:** Comparison of QCA between baseline and 5-year follow-up (N = 25)

QCA (mean ± SD)	Baseline	5 years	P-value^a^
Scaffold length (mm)	19.80 ± 8.45	19.90 ± 8.51	0.64
Minimal lumen diameter (mm)	2.47 ± 0.34	2.36 ± 0.39	0.13
Mean lumen diameter (mm)	2.9 ± 0.36	2.94 ± 0.42	0.55
Proximal RVD (mm)	3.13 ± 0.41	3.10 ± 0.42	0.71
Distal RVD (mm)	2.75 ± 0.55	2.75 ± 0.63	0.89
Mean RVD (mm)	2.93 ± 0.39	2.93 ± 0.44	0.98
Diameter stenosis (%)	15.6 ± 15.9	18.9 ± 8.3	0.05
Late lumen loss (mm)	–	0.11 ± 0.35	–

*QCA* quantitative coronary angiography, *SD* standard deviation, *RVD* reference vessel diameter

^a^Paired t-test

**Fig. 4:**
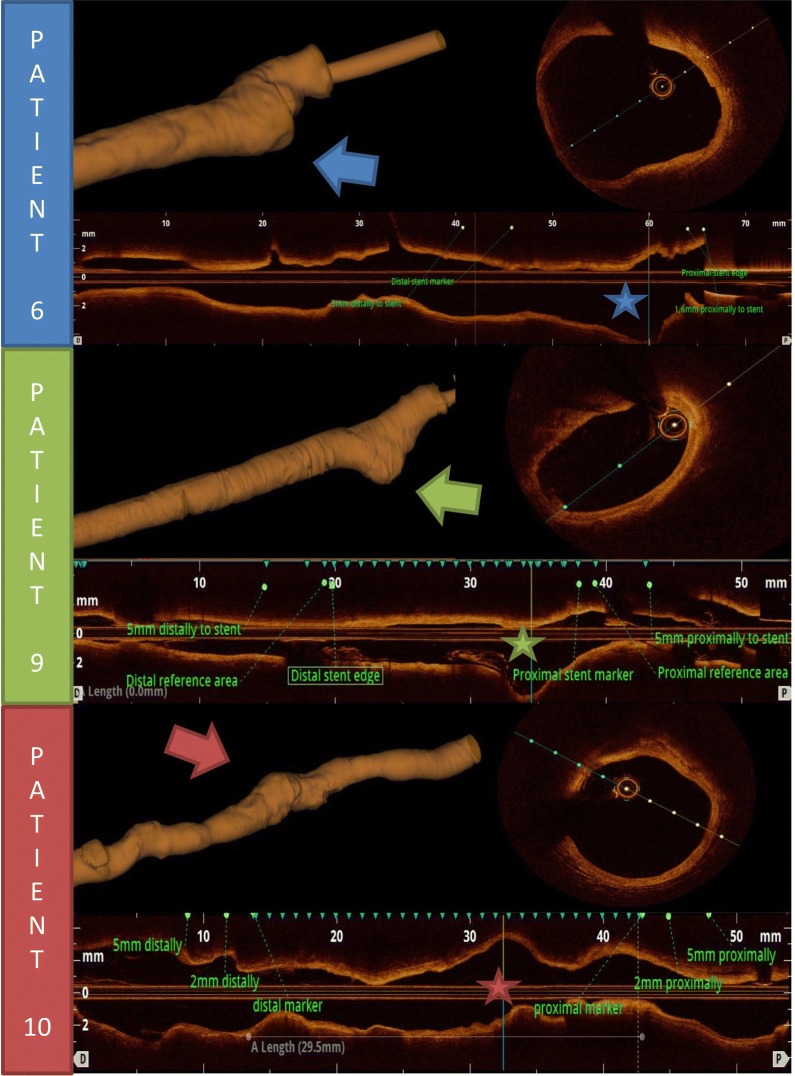
OCT analysis of three patients with coronary artery aneurysm detected after 5 years post BRS Absorb implantation in STEMI. Patients are numbered. Left columns present three-dimensional OCT reconstruction, aneurysms are marked by arrows. Right column shows cut through the maximum vessel dilatation, the cut is marked by asterisk on the longitudinal OCT image. *BRS* bioresorbable scaffold, *OCT* optical coherence tomography, *STEMI* ST-elevation myocardial infarction

Baseline OCT was available in 15 patients. All 25 patients had OCT performed at the time of five-year follow-up, but three patients were excluded from analysis due to sub-optimal image quality precluding clear identification of scaffold markers. Table 4 presents paired comparison of 14 patients who had both baseline and five-year OCT available for analysis. There is no difference in reference vessel dimensions. Scaffold mean area at baseline is similar to lumen mean area after five years; 8.46 ± 1.55 mm^2^ versus 7.9 ± 2.12 mm^2^ (P = 0.13). Scaffold and lumen mean diameters at baseline and after five years are nearly identical. There were no scaffold struts and no thrombi present after five years. Analysis of segments with three new coronary artery aneurysms showed normal arterial three-layer structure and did not demonstrate any thrombi or dissections (Fig. 4).

**Table 4:** Comparison of OCT between baseline and five-year follow-up (N = 14)

OCT parameter (mean ± SD)	Baseline	5 years	P-value^a^
Proximal reference area (mm^2^)	7.69 ± 1.70	7.76 ± 1.93	0.79
Proximal reference diameter (mm)	3.10 ± 0.35	3.12 ± 0.41	0.75
Distal reference area (mm^2^)	5.61 ± 2.15	6.20 ± 2.77	0.28
Distal reference diameter (mm)	2.61 ± 0.52	2.74 ± 0.61	0.25
Scaffold/lumen mean area (mm^2^)	8.46 ± 1.55	7.9 ± 2.12	0.13
Scaffold/ lumen mean diameter (mm)	3.25 ± 0.30	3.22 ± 0.49	0.73
Minimal lumen area (mm^2^)	–	5.23 ± 1.85	–
Minimal lumen diameter (mm)	–	2.56 ± 0.41	–
Stent length per OCT (mm)	21.96 ± 8.39	22.60 ± 10.63	0.6
Mean number of frames	21.43 ± 8.88	22.93 ± 11.01	0.31
Mean number of struts	190.21 ± 86.91	0	–
Mean number of struts per frame	8.83 ± 1.08	0	–

*OCT* optical coherence tomography, *SD* standard deviation

^a^Paired t-test

## Discussion

Our study reports on the largest cohort of STEMI patients treated with BRS with five-year clinical and imaging follow-up available. The main findings are the following:

1. The primary clinical endpoint at five years occurred in 12.6% with definite/probable BRS thrombosis rate of 2.5%. This clinical data should be however regarded in light of not completed 5 year clinical follow-up of the whole study group.
2. Angiographic late lumen loss five years post BRS implantation was only 0.11 ± 0.35 mm with binary restenosis rate of 0%. Complete scaffold resorption at 5-year follow-up is confirmed by OCT and the mean lumen area remained stable in comparison to baseline.
3. Three patients have developed small coronary artery aneurysm in the segment treated by Absorb™ BRS.

We report good clinical results till five-year follow-up with only 12 months duration of dual antiplatelet therapy. This can be explained by several factors: very selected population of patients in our study due to previously published [9] inclusion and exclusion criteria of Prague-19 study (in brief patients with heart failure, poor prognosis, high risk of bleeding and long or calcified lesion were excluded); Absorb™ BRS expansion might be better in soft thrombotic plaques in STEMI patients and favourable vessel healing already 6 months after Absorb™ BRS implantation during primary PCI has been documented previously [16, 17]. Our results are in agreement with two randomized studies (TROFI II, ISAR-Absorb MI) and one propensity matched analysis of BRS versus metallic drug-eluting stents, which showed no significant clinical differences in mid-term (maximum 2 years) outcome in STEMI patients [18–21]. Importantly, we did not observe any cardiac events related to discontinuation of dual anti-platelet therapy after 12 months post BRS implantation.

Several studies have reported angiographic and intravascular imaging results 5 years post Absorb™ BRS implantation [6–8]. None of the published studies included patients with STEMI, but the first case report 4 years post primary PCI has been described [22]. Angiographic late lumen loss in patients with stable coronary lesions ranges from 0.13 mm to 0.22 mm, which is similar to our results. This is also similar to our previously published (different patient cohort) late lumen loss of 0.2 mm after three years [10]. The stable lumen area/diameter measured by both QCA and OCT after complete strut resorption provides proof, that the mechanical support provided by permanent metallic stents is not needed to maintain vessel patency in the long-term. However, the first case of incomplete absorption even after 5 years has been reported and delayed endothelization might be a contributing factor [23]. It is therefore reassuring that we did not detect any discernible strut remnants. The permanent presence of struts after metallic stent implantation can be associated with malapposition in 65% and 32% of stents detected by OCT immediately post procedure and after 8 months, respectively [24]. Incomplete stent apposition present immediately post implantation can resolve in 74% of patients (everolimus-eluting stent) [25] and, on the other end, late-acquired malapposition can develop in 17% of stents (sirolimus or paclitaxel-eluting stents) [24]. The clinical importance of late stent malapposition is however not clear, recently published article did not find any association with unfavourable clinical events after 8 years of follow-up [26].

Coronary artery aneurysms after drug-eluting metallic stent implantation are related to dissections, deep arterial wall injury or arterial wall inflammation in response to the drug, polymer or metal. Coronary artery aneurysm incidence after sirolimus or paclitaxel eluting metallic stents is between 0.2 and 2.3% [12], the clinical importance is not clear, it may be associated with worse outcome according to recent and large study [27]. Coronary artery aneurysms after Absorb™ BRS implantation have been observed in 3% of patients [28]. One retrospective study suggests that lesion preparation with cutting balloon is associated with coronary aneurysm formation in polymeric BRS implantation [29]. Aneurysms are dynamic and can recede spontaneously [30]. All three aneurysms reported in our study are small and without any sign of arterial wall injury in the moment of the study. Further study of coronary aneurysms post BRS implantation would require multi-centre cooperation with pooling of data.

## Limitations

There are obvious limitations due to the single-arm non-randomized study design with significant selection bias. All analysis was limited to Absorb™ BRS patients with no direct comparator. The moderate number of enrolled patients does not allow us to reach any definitive conclusions on clinical outcomes. We present 5 year clinical data of only 81 out of 134 patients (60%) with implanted BRS, thus it should be regarded as subanalysis of the PRAGUE-19 study. However, this article was mainly focused on 5 year imaging and final clinical data are planned to be published in 2021. The invasive assessment is also limited by small number of studied patients but represents the only available scientific data on long-term follow-up after Absorb™ BRS use in STEMI patients and the number of patients is similar to other studies with long-term invasive follow-up of BRS technology. All analysis was performed on-site and not in the independent core-laboratory. The inclusion criterium (invasive study at five-year follow-up) could introduce inclusion bias, this is however clinically unlikely as the more symptomatic patients should be more likely to agree to repeat coronary angiography.

Our results should be interpreted as hypothesis-generating and confirmed by larger, randomized studies with long term follow-up. Nevertheless, stable vessel architecture after BRS resorption that we have observed in our imaging substudy, is promising-proof of concept for future generation of BRS with shorter resorption process. In the era of development of new stents technologies, careful imaging assessment in the long-term follow studies next to large cohort clinical assessment is essential to translate new technologies into daily clinical practice.

## Conclusions

This Prague-19 substudy analysis of STEMI patients treated with Absorb™ BRS presents the first clinical and imaging results after five years of follow-up. Angiographic late lumen loss is very low, and OCT confirmed complete resorption of BRS struts. STEMI might represent very suitable setting for further development of BRS technology.

### Impact on daily practice

Results of Prague-19 study 5-year follow-up together with recently published ISAR-Absorb MI trial one-year safety data could lead to clinical testing of new generation BRS in patients with STEMI.

## Data Availability

The datasets during and/or analysed during the current study available from the corresponding author on reasonable request.

## Abbreviations

BRS: bioresorbable scaffold
MI: myocardial infarction
MLD: minimal lumen diameter
OCT: optical coherence tomography
PCI: percutaneous coronary intervention
QCA: quantitative coronary angiography
RVD: reference vessel diameter
STEMI: ST elevation myocardial infarction
TVR: target vessel revascularization
